# Measuring moderate-intensity walking in older adults using the ActiGraph accelerometer

**DOI:** 10.1186/s12877-016-0380-5

**Published:** 2016-12-08

**Authors:** Anthony Barnett, Daniel van den Hoek, David Barnett, Ester Cerin

**Affiliations:** 1 Institute for Health & Ageing, Australian Catholic University, Melbourne, Victoria Australia; 2 School of Exercise and Nutrition Sciences, Deakin University, Burwood, Victoria Australia; 3 School of Public Health, The University of Hong Kong, Hong Kong, Hong Kong SAR People’s Republic of China

**Keywords:** Physical activity, MET, Energy expenditure, Resting metabolic rate, Measurement, Vector magnitude

## Abstract

**Background:**

Accelerometry is the method of choice for objectively assessing physical activity in older adults. Many studies have used an accelerometer count cut point corresponding to 3 metabolic equivalents (METs) derived in young adults during treadmill walking and running with a resting metabolic rate (RMR) assumed at 3.5 mL · kg^−1^ · min^−1^ (corresponding to 1 MET). RMR is lower in older adults; therefore, their 3 MET level occurs at a lower absolute energy expenditure making the cut point derived from young adults inappropriate for this population. The few studies determining older adult specific moderate-to-vigorous intensity physical activity (MVPA) cut points had methodological limitations, such as not measuring RMR and using treadmill walking.

**Methods:**

This study determined a MVPA hip-worn accelerometer cut point for older adults using measured RMR and overground walking. Following determination of RMR, 45 older adults (mean age 70.2 ± 7 years, range 60–87.6 years) undertook an outdoor, overground walking protocol with accelerometer count and energy expenditure determined at five walking speeds.

**Results:**

Mean RMR was 2.8 ± 0.6 mL · kg^−1^ · min^−1^. The MVPA cut points (95% CI) determined using linear mixed models were: vertical axis 1013 (734, 1292) counts · min^−1^; vector magnitude 1924 (1657, 2192) counts · min^−1^; and walking speed 2.5 (2.2, 2.8) km · hr^−1^. High levels of inter-individual variability in cut points were found.

**Conclusions:**

These MVPA accelerometer and speed cut points for walking, the most popular physical activity in older adults, were lower than those for younger adults. Using cut points determined in younger adults for older adult population studies is likely to underestimate time spent engaged in MVPA. In addition, prescription of walking speed based on the adult cut point is likely to result in older adults working at a higher intensity than intended.

## Background

Worldwide, the number of older adults is growing rapidly, with an expected increase in those 60 years or older from ~810 million (11.7%) in 2013 to more than 2 billion (21.1%) in 2050 [1]. From an individual and community perspective, it is paramount to encourage lifestyle behaviours that will maintain health, functionality and independent living in older adults. The World Health Organisation (WHO) recommends engaging in moderate-to-vigorous intensity physical activity (MVPA), which has been positively associated with numerous health outcomes in older adults including lower rates of all-cause mortality, type 2 diabetes, stroke, coronary heart disease, high blood pressure and some cancers [2, 3]. It may also improve cognitive function and delay the onset of cognitive disease [4].

Given that physical activity (PA) recommendations for health benefits are intensity specific, it is important to accurately differentiate light intensity PA from MVPA. PA intensity is typically categorised as light, moderate and vigorous based on metabolic equivalents (METs); one MET being the resting metabolic rate (RMR) and assumed to be a $\dot{V}{O}_2$ of 3.5 mL · kg^−1^ · min^−1^ [5]. An intensity of 3 METs represents the commonly-accepted cut-off value between light and moderate intensity PA [5]. At present, the adult standard RMR above and associated MET levels for categorising PA intensity are commonly used in observational studies of older adults (e.g., [6–9]). However, it is well established that RMR decreases with age [10, 11]. This lower individual energy expenditure associated with RMR has implications for the determination of relative rate of energy expenditure during PA in older adults. With a lower RMR, the energy expenditure in absolute terms associated with a given MET activity intensity threshold will be lower in older compared to younger adults. With MET-based PA intensity values computed using the conventional RMR of 3.5 mL · kg^−1^ · min^−1^, older adults would be working at higher relative intensities than assumed and their time spent in PA above activity intensity thresholds would be underestimated [12].

Hip-worn accelerometers, used to objectively measure PA, can relatively accurately quantify PA intensity of ambulatory activities (e.g., walking) [13]. They are particularly appropriate for assessing PA in older adults as they require no input from the participant over the collection period and eliminate bias related to subjective recall of past events associated with estimation of PA via questionnaires [14], an ability that can decline with ageing. Accelerometer data can be quantified as counts-per-minute with established count cut points and ranges categorizing light, moderate or vigorous PA intensity. The ActiGraph is the most widely used accelerometer in PA research [14].

Derivation of cut points involves establishing relationships between energy expenditure and accelerometer counts. Several studies determining ActiGraph PA cut points in older adults included a variety of activities such as resting, household chores or physical exercises performed at a single, often light, intensity [15, 16]. Many typically occur infrequently or on a weekly basis (e.g., sweeping or dusting), therefore likely making a minimal contribution to average daily energy expenditure. Also, the effect of performing these activities at different intensities (e.g., sweeping at different speeds) on energy expenditure and accelerometer counts has not been investigated. Furthermore, it seems that increases in PA in older adults are less likely to come from increases in the volume of these activities (e.g., household chores) than from activities of preference, such as walking [13, 17, 18]. Determining ActiGraph moderate-intensity PA cut points from protocols involving a large proportion of infrequently performed activities or tasks consisting of primarily upper limb movements (e.g., dusting, washing dishes), which waist-worn accelerometers cannot accurately assess, rather than ambulatory activities (e.g., walking) is problematic as it is likely to result in too low cut point values.

Also, previous studies determined cut points using treadmill walking [12, 15, 16, 18, 19] which produces lower ActiGraph counts and higher energy expenditure for a given walking speed compared to overground walking, therefore overestimating energy expenditure and free-living walking speed predicted by accelerometer [20]. Lastly, while not problematic in large population studies, the levels of utility and generalisability of accelerometer cut points for the quantification of PA intensity in older adults remain unclear. High inter-individual cut point variability would indicate that individual specific cut points would be preferable to a ‘general’ , group-derived cut point when monitoring and comparing the PA and related energy expenditure levels in individuals or small groups of participants. Conditions such as walking-related balance difficulties and declining muscle strength may modify gait speed and efficiency with ageing [21, 22]. Resulting changes in gait pattern may introduce greater variations in movement and, therefore, accelerometer counts during walking. In a sedentary population of older adults selected based on being sedentary and at risk for mobility disability, high variability in mean accelerometer count was observed during supervised walks at a participant defined ‘moderately hard’ intensity [23]. While it is difficult to separate the variability due to individual differences in accelerometer counts associated with a specific intensity cut point and variability due to individual perception of that intensity, these data suggest high levels of inter-individual cut point variability. Inter-individual variability of the objectively measured 3 MET MVPA cut point in older adults has yet to be quantified.

A recent review of ActiGraph accelerometry in older adults found that 35 of 53 articles using vertical axis (VA) accelerometer counts to measure MVPA used cut points in the range of 1952 to 2020 counts · min^−1^ to define the lower limit of MVPA [24]. This range is delimited by cut points determined in younger adults [25, 26]. As older adults have a lower RMR and higher energy expenditure when walking at a given speed compared to younger adults [27], older adult-specific accelerometry cut points should be applied, rather than those derived from younger populations.

Few MVPA cut point calibration studies in older adults have used measured RMR. To account for the lower RMR in older adults, some non-walking-specific cut point studies have used investigator-selected RMR or MVPA cut point values. A recent study in older women undertaking a variety of activities chose an energy expenditure of 3 mL · kg^−1^ · min^−1^ based on sitting quietly watching a DVD for RMR [28]; incidentally, this was close to the RMR range of 2.7 to 2.9 mL · kg^−1^ · min^−1^ reported in studies of older adults with similar mean ages [11, 12, 29, 30]. Another study based the MVPA cut point on a walking speed of 3.2 km · hr^−1^, corresponding to an energy expenditure of 13 mL · kg^−1^ · min^−1^ in the study population [18]. This intensity is equivalent to 3.7 METs using the adult standard RMR of 3.5 mL · kg^−1^ · min^−1^ or 4.6 METs for an RMR of 2.8 mL · kg^−1^ · min^−1^, the middle of the range of older adults RMRs mentioned above, and resulted in a MVPA cut point of 1041 counts · min^−1^ [18]. Both are higher than the accepted MVPA cut point intensity of 3 METs. The two previous studies that used measured RMR when examining the relationship between VA accelerometer (ActiGraph GT3X) counts and the 3 MET cut point specifically for walking in older adults have limitations [12, 31]. These included small sample sizes (20 and 15 older adults) and the use of treadmill walking in the determination of speed, energy expenditure and accelerometer count relationships [20]. Also, ordinary linear regression models on pooled data were used to determine the relationship between counts and energy expenditure. This commonly used method does not account for the assumption of independency of observations being violated when there are multiple sets of data points (speeds) per participant, and may lead to spurious results.

Accelerometer data is typically processed as counts per unit of time. Older piezoelectric based accelerometers were limited to one axis and recorded counts associated with vertical displacement, that is, VA counts. The introduction of piezoelectric based accelerometers has allowed the collection of data across three axes and vector magnitude (VM) (the square root of the sum of the squares of the vertical, anterior-posterior and medial-lateral axes) accelerometer counts have also been used in the measurement of PA intensity. While the majority of population studies use VA counts, it is unclear at present whether MVPA cut point determination using a single axis or multiple axes is superior [32, 33].

Based on measured RMR and sound methodology, the primary aim of this study was to determine the overground walking MVPA accelerometer count (VA and VM) and speed cut points for older adults; walking being the most popular PA among older adults [17, 18]. The secondary aim was to quantify inter-individual variability in older adult MVPA cut points.

## Methods

### Participants

Forty-five older adults (age 70.2 ± 7 years, range 60–87.6 years) participated in the study. Participants were recruited via an online event-advertising site, older adult newspapers and word-of-mouth. Inclusion criteria were, ≥60 years and able to walk unaided. Exclusion criteria were any reported health or medical condition limiting ability to undertake light-to-moderate walking and medically advised presence of diabetes. Diabetics were excluded due to possible implications of the pre-RMR fast, such as hypoglycaemia and hyperglycaemia. Following reading a plain language statement outlining the study, asking any follow-up questions and satisfying the above criteria, participants gave written informed consent. This study was approved by Deakin University - Health Ethics Advisory Group.

### Study design

Participants were asked to fast, with the exception of water, for a minimum of 5 h prior to arrival at the clinic, abstain from caffeine overnight, abstain from smoking and alcohol for at least the preceding 2 h, not undertake moderate intensity exercise for the preceding 2 h and not undertake vigorous activity for the preceding 14 h [34]. Anthropometric data were measured and RMR determined. Participants then undertook an outdoor graded overground walking protocol on a flat concrete surface with accelerometer count and metabolic rate determined at five walking speeds.

### Resting metabolic rate

In a quiet, 22 °C room, participants spent 20 min in a recumbent posture, upper body at an angle of 30° and pillow under the head for comfort. During the final 10 min, expired gas was continuously analysed using a mobile breath-by-breath gas analysis system (MetaMax® 3B CORTEX Biophysik GmbH, Leipzig, Germany). Data were stored in 5-s intervals and RMR defined as the lowest 5-min moving average. The MetaMax® 3B has previously been used in PA studies involving measurement of RMR [35, 36]. As the turbine and volume sensor have a specification of 7 ml resolution, potential small tidal volumes associated with the low ventilation flow rates that older adults may exhibit during an RMR determination can be accurately measured.

### Walking protocol

A 200 m familiarization walk with all equipment and a minimum 5 min recovery period preceded the protocol. The walking protocol was a modification, using slower walking speeds and walking up-and-back over 100 m, of one previously used for adults [20]. Slower walking speeds were included to ensure the range encompassed the 3 MET cut point and provided walking speeds possibly representative of the ambulatory component of day-to-day activities. Participants completed the walk at increasing speeds with data collected via an ActiGraph GT3X+ accelerometer (ActiGraph, Pensacola, FL), Global Positioning System (GPS) monitor, (Qstarz BT-Q1000P GPS data logger, Qstarz International Co., Taipei, Taiwan) and the portable metabolic system. The accelerometer and GPS monitor were worn on the right hip. The GT3X+ low frequency extension option (GT3X + LFE), which increases sensitivity to very low amplitude activities and suited for older adults who may move slowly or take very light steps, was used for all accelerometer data. It has also been recommended due to yielding more comparable data to older accelerometer models than the GT3X+ with the normal filter [37]. Data were collected at 5-s intervals with all measurement apparatus synchronised to atomic clock time. During the walk, the participant walked beside the researcher who regulated the speed and distance via a GPS monitor (Forerunner 201 GPS monitor, Garmin Ltd, Olathe, KS). This GPS monitor has been shown to be accurate for monitoring speed over a given distance in a relatively small, open-sky environment [38]. Total walking distance was 1000 m, with speeds of 1.6, 2.2, 2.8, 3.4 and 4 km · hr^−1^, for 100, 200, 200, 200 and 300 m respectively. Participants were advised they could withdraw from the data collection procedure at any time.

### Energy expenditure

Prior to data collection for each participant, the portable oxygen analyser was calibrated according to the manufacturer’s guidelines (Calibration Manual 931-00-264/Revision a/2014-03-06, CORTEX Biophysik GmbH, Leipzig, Germany). During data collection, the participants wore a face mask with flow meter over the nose and mouth and connected to the mobile gas analyser via the sample line. To eliminate the effect of the weight of the gas analyser on energy expenditure during the walking protocol, it was worn by the researcher walking beside the participants. $\dot{V}{O}_2$ was measured using breath-by-breath mode and, to match the time periods across devices, data were stored in 5-s intervals.

### Data analysis

The last 2 min at each of the five walking speeds were used to determine $\dot{V}{O}_2$ and accelerometer counts for that speed. As walking speed can vary slightly during each walking stage, speed matching the time period for $\dot{V}{O}_2$ and accelerometer count determination within each stage was determined from Qstarz GPS monitor data. As VA and VM accelerometer counts have been used in measurement of PA intensity, both were examined in the current study.

As the assumption of independency of observations was violated due to having five sets of data points (speeds) per participant, linear mixed (LM) models with random intercept and slopes were used to determine the VA and VM counts and walking speed associated with an energy expenditure of 3 METs for the ‘average’ subject. LM models also allow the inclusion of covariates such as gender, age and BMI, provide information on inter-individual variability in cut points and enable the determination of the most parsimonious regression model (e.g., linear term of METs vs. higher-order polynomials of METs).

LM models were run with METs centred at 3.0 so that the average participant’s VA and VM counts and walking speed cut points (and their 95% confidence intervals (CI)) for moderate-intensity PA (i.e., 3 METs) could be derived from the point estimate of the random intercept of the respective LM models. The inter-individual variability in a specific cut point was quantified by the standard deviation of the random intercept. For each, several models of increasing complexity were estimated, starting with models with fixed intercept and linear fixed slope for METs through models with random intercept and random cubic polynomial slopes for METs. Final model selection was based on the likelihood ratio test. After determining the best fitting model for a particular variable, we examined the extent to which each of gender and mean-centred age, height, weight and BMI explained VA, VM and walking speed variability in the intercept by adding each of these to the models. Accuracy of prediction of METs by VA count and VM was compared using the Akaike information criterion (AIC) from the best fitting models excluding demographic covariates. Analysis was undertaken using R 3.2.3 [39] with packages lme4 for LM models [40].

## Results

Participant characteristics are presented in Table 1. Mean RMR was 2.8 ± 0.6 mL · kg^−1^ · min^−1^. The gender mean values of resting metabolic rate for our participants were not significantly different: F = 2.78 mL · ml-1 · kg −1, M = 2.93 mL · ml-1 · kg −1, *p* = 0.36 with Cohen’s d estimated to be 0.28 (small effect size in favour of males with slightly higher RMR). Others have also found no significant difference between the weight-related RMR values of male and female older adults [11, 30].

**Table 1:** Participant demographic characteristics (*N* = 45)

Characteristic	Value	Range
Age (years) mean ± SD	70.2 ± 7	60–87.6
Sex (female) n (%)	29 (65)	
Height (m) mean ± SD	1.69 ± 0.09	1.55–1.94
Weight (kg) mean ± SD	78.6 ± 14.0	54.0–104.1
Body mass index (kg · m^−2^) mean ± SD	27.4 ± 4.0	20.3–38.5
Resting metabolic rate (mL · kg^−1^ · min^−1^) mean ± SD	2.8 ± 0.6	1.9–4.4

**Fig. 1:**
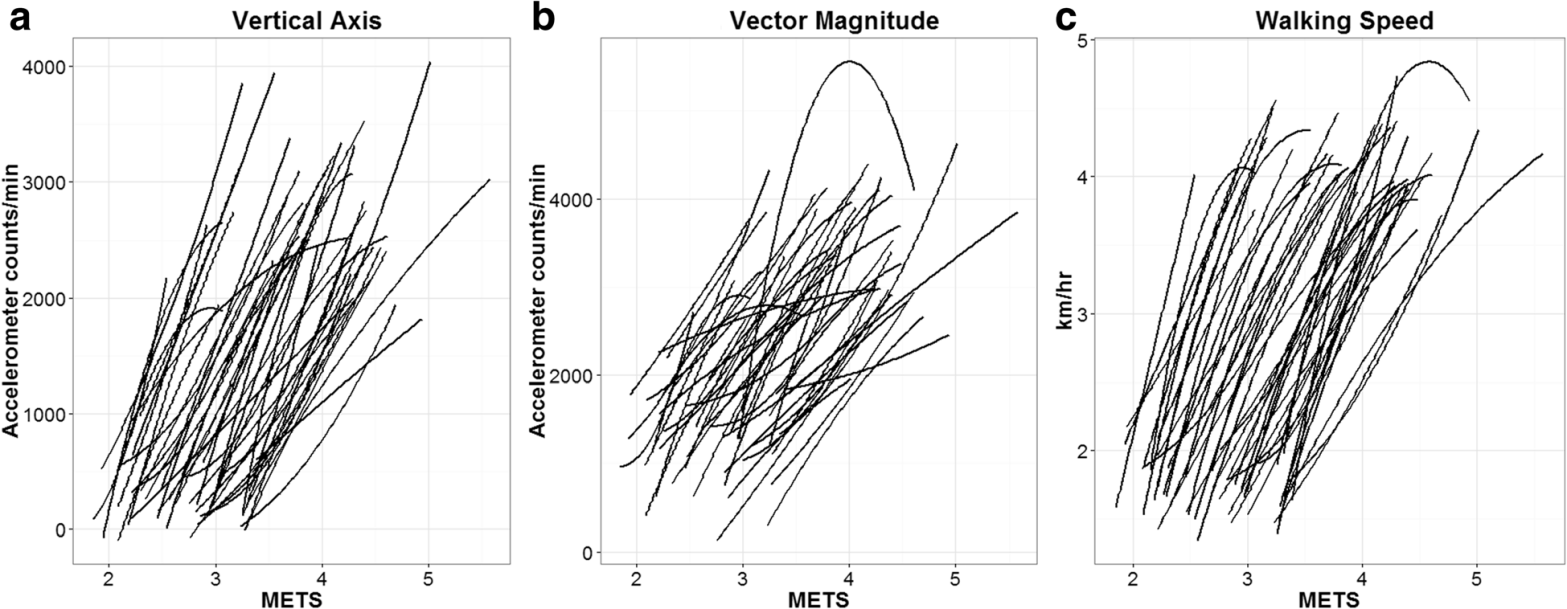
Individual participant VA (**a**), VM (**b**) and walking speed (**c**) as smoothed functions of METs

**Fig. 2:**
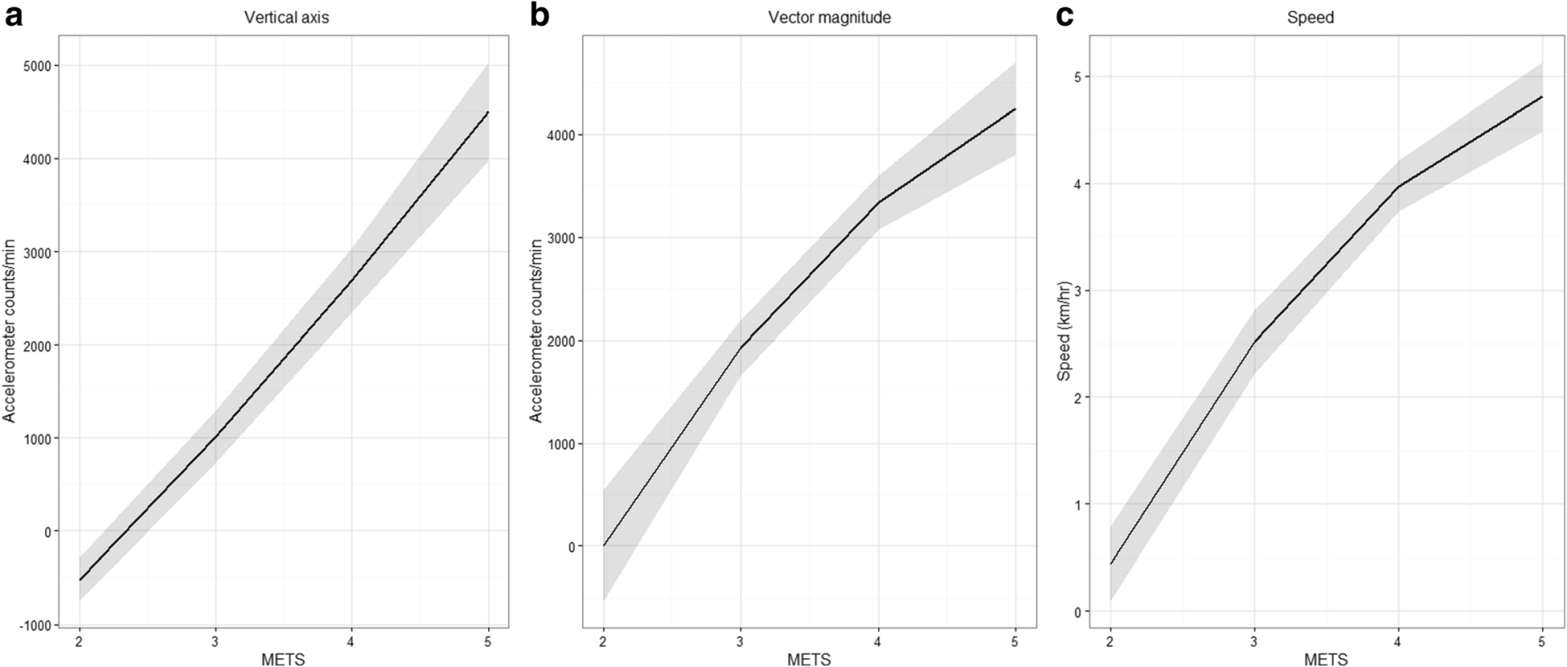
Quadratic relationships of VA (**a**), VM (**b**) and walking speed (**c**) with METs for the average participant (95% CI *grey shade*)

Average walking speeds (SD) during the last 2 min of each walking speed were 1.7 ± 0.2, 2.2 ± 0.2, 2.8 ± 0.2, 3.5 ± 0.3 and 4.1 ± 0.3 km · hr^−1^. There was considerable inter-individual intercept and slope variation for VA, VM and walking speed as predicted by METs (Fig. 1). Models with random intercept and slope including a linear and a quadratic term of METs provided the best fit for VA, VM and walking speed (Fig. 2). Table 2 reports the LM model estimates of regression parameters (intercept, slopes, random effects) for each variable. MVPA cut points (95% CI), represented by the point estimates of the intercepts of the models in Table 2, were: VA 1013 (734, 1292) counts · min^−1^, VM 1924 (1657, 2192) counts · min^−1^, walking speed 2.5 (2.2, 2.8) km · hr^−1^. The inter-individual variability in cut points, corresponding to the random intercept standard deviations, were: VA 941 counts · min^−1^, VM 889 counts · min^−1^, walking speed 1.0 km · hr^−1^. That is, 67% of older adults would be expected to have MVPA thresholds in the range of 72 to 1954 VA counts · min^−1^, 1035 to 2813 VM counts.min^−1^ and 1.5 to 3.5 km · hr^−1^ for walking speed. The fit of all three models improved with BMI as a covariate, but not by the inclusion of age, height, weight or gender independently or age or gender in association with BMI. Inclusion of BMI led to a reduction in inter-individual variability of cut points of 19.3% for VA, 20.3% for VM and 18.2% for walking speed. When predicting METs, model fit was better with VA as a predictor (AIC = 61.377) compared with VM (AIC = 157.171).

**Table 2:** Linear mixed model estimates of regression parameters

Variable	Model^a^	Intercept (95% CI) (MVPA cut point)	METs (linear term) (95% CI)	METs (quadratic term) (95% CI)	BMI (95% CI)	Intercept SD (inter-individual variability in MVPA cut point)
VA (counts · min^−1^)	1	1013 (734, 1292)	1062 (902, 1221)	71 (−51, 193)	-	941.3
	2	1015 (763, 1267)	1622 (1435, 1793)	70 (−50, 190)	−73 (−128, −18)	845.8
VM (counts · min^−1^)	1	1924 (1657, 2192)	1665 (1363, 1967)	−251 (−450, −51)	-	888.6
	2	1931 (1690, 2172)	1656 (1350, 1962)	−181.1 (−384, 22)	−81 (−140, −22)	793.2
Walking speed (km · hr^−1^)	1	2.5 (2.2, 2.8)	1.8 (1.6, 1.9)	−0.3 (−0.4, −0.2)	-	0.9920
	2	2.5 (2.3, 2.8)	1.7 (1.6, 1.9)	−0.2 (−0.4, −0.1)	0 (−0.1, 0)	0.8973

^a^Model 1 = quadratic equation with random slope and intercept with METs (centred at 3 METS) as the predictor, Model 2 = model 1 with the addition of BMI (centred at the mean value, 27.4 kg · m^−2^) as a covariate

*MVPA* moderate-to-vigorous intensity physical activity, *CI* confidence intervals, *SD* standard deviation, *VA* vertical axis, *VM* vector magnitude, *BMI* body mass index

## Discussion

### Primary aim: determination of the walking MVPA accelerometer count and speed cut points for elderly based on measured RMR

As hypothesised, the vertical axis cut point of 1013 counts · min^−1^ was substantially lower than 1952 counts · min^−1^; the most commonly used cut point in older adults [24] and determined in a sample of adults with mean age of <25 years [25]. The difference is likely due to a number of influences, one being the mean RMR of 2.8 mL · kg^−1^ · min^−1^ found in this study compared to the RMR of 3.5 mL · kg^−1^ · min^−1^ used by Freedson et al. [25]. This equates to 20% lower absolute energy expenditure at 3 METs in this population. The RMR for our participants was in the range of 2.7 to 2.9 mL · kg^−1^ · min^−1^ reported in studies of similarly aged older adults [11, 12, 29, 30] and in line with the expected decrease in RMR with ageing. Coupled with energy expenditure at a given walking speed being higher in older adults [27], which would be expected to result in a lower accelerometer count for a given level of energy expenditure, the lower 3 METs accelerometer count observed in our study population is not surprising. Age has also been shown to be associated with increased energy expenditure and reduced accelerometer counts within a sample of obese adults (mean age 43.2 years) [41]. Based on a single self-paced 400 m walk and selected RMR of 3 mL · kg^−1^ · min^−1^, Evenson et al. found a lower MVPA VA cut point of 944 counts · min^−1^ in older women using the ActiGraph GT3X + LFE accelerometer and receiver operating characteristics (ROC) curve analysis [28]. We have found only one other study for which comparison with our VA data could be justified. In this study, which used measured RMR and LM models, the MVPA VA cut point in middle-aged to old obese/overweight type 2 diabetes mellitus patients (mean age 62.5 years, BMI 30 kg∙m^−2^) was 1240 counts · min^−1^ [15]. Considering the use of overground rather than treadmill walking in our study and the higher energy expenditure for a given walking speed/accelerometer count with aging, there appears to be reasonable concordance with our older aged participants’ cut point of 1013 counts · min^−1^.

The MVPA VM cut point in the current study was 1924 counts.min^−1^. A similar cut point of 1776 counts · min^−1^ based on a 400 m walk was determined in older women using the Actigraph GT3X + LFE and ROC curve analysis [28]. No other studies to date appear to have determined MVPA VM cut points for walking in older adults.

According to the Compendium of Physical Activities (5), the walking speed on a level surface associated with 3 METs in adults is 4 km · hr^−1^, where 1 MET = 3.5 mL · kg^−1^ · min^−1^. However, this does not appear to apply to all adult populations. A mean 3 MET walking speed of 2.6 km · hr^−1^ based on individual RMRs has been reported in obese-to-severely obese adults (mean age 43 years, mean BMI 40 kg · m^−2^) [42]. For the older adults in our study, the 3 MET walking speed (the MVPA cut point) determined using measured RMR was substantially lower at 2.5 (95% CI 2.3–2.8) km · h^−1^. Advising or monitoring walking intensity in older adults using the 4 km · hr^−1^ speed typically associated with the MVPA cut point in younger adults would involve older adults walking at a much higher intensity than desired. Higher absolute intensities than necessary may also result in lower compliance to activity guidelines [43]. No other studies reporting walking speed at 3 METs in older adults could be found.

### Secondary aim: examine inter-individual variability in MVPA cut points

To the best of our knowledge, this is the first accelerometer MVPA (3 MET) cut point calibration study to quantify inter-individual variability in cut points in older adults. We found substantial inter-individual variability in the MVPA cut point for VA, VM and walking speed (Table 2). MVPA cut points are largely used in the determination of population effects of PA on health conditions and the appropriate MVPA dose for these conditions. While group cut points can be used in large, population-based studies, it is important to be mindful of the large inter-individual variability associated with PA estimates based on accelerometer cut point in older adults when interpreting findings. When the goal is to accurately estimate energy expenditure or time spent above the MVPA cut point, the observed high inter-individual variability suggests that the use of individually determined MVPA cut points would be preferable in small-sample and clinical studies as well as individual applications, as recently noted by Rejeski et al. with respect to sedentary older adults at risk of mobility disability [23]. As the presented methodology for determination of an individual MVPA cut point is not practical for outside of a research setting, reliable and valid estimations are needed. While the Borg Rating of Perceived Exertion scale, frequently used as a subjective measure of PA intensity [44], also exhibits some inter-individual variability in older adults [45], its concordance with individual MVPA cut points in older adults could be investigated. Alternatively, identification of person characteristics in addition to BMI that explain the variance in individual cut points may enable the determination of a suitable estimation equation.

### Strengths and limitations

For reasons already outlined above, the current study used LM models for data analysis, overground rather than treadmill walking, measured RMR and determination of inter-individual differences in cut points. Calibration studies have typically derived cut points using ordinary linear regression on pooled data, LM models or ROC curves. As previously mentioned, ordinary linear regression fails to account for the independency of observations being violated when there are multiple sets of data points (speeds) per participant and may lead to spurious results. Both ordinary linear regression and ROC do not provide information on inter-individual variability in cut points. Also, ROC does not allow the introduction of covariates as predictors, and can result in implausible cut points unlikely to be valid, as well as substantially different cut points based on sensitivity and specificity compared to those based on accuracy, hence making the choice of an appropriate cut point difficult [36]. Therefore, we believe that LM models are the most informative analytical method for cut point determination.

The study has a number of limitations, including the sample of older adults with a mean age of 70 years and able to walk unaided not being representative of all older adults. Old-old adults are likely to have lower RMR and a greater energy cost during walking [22]. Those with gait problems are also likely to have a greater energy cost during walking as well as varying gait patterns which may affect the accelerometer count to energy consumption relationships. Second, wearing the mask for expired air analysis may have affected gait and, therefore, accelerometer counts. However, measurement of energy expenditure associated with slow overground walking in older adults has been shown to be reliable [46] and, in a small subsample of a study in adults, there was no difference in VA counts during walking with and without a portable gas analyser [20]. Third, the cut points determined apply to walking. Other activities will have different MVPA cut points. Having a mix of different activities in the calibration procedure will result in cut points specific to that mix. This would potentially result in a large number of cut points based on different activity and activity intensity combinations. It would seem that changes or differences in energy expenditure across older adults are more likely to be associated with walking than regular household activities, often undertaken at low weekly frequency. Therefore, we preferred to focus on walking, the most common PA in older adults [13, 17, 18], over a range of intensities. As its sensitivity to very low frequencies is appropriate for older adults, the ActiGraph GT3X + LFE was used in this study. In younger adults, the use of the LFE with the ActiGraph GT3X+ has been shown to produce output comparable to the 7164 model [32], the output of which was not significantly different from the three versions of the GT1M during walking and running [47]. Therefore, while future studies using the determined cut points should ideally use the GT3X + LFE, they may also be suitable for data collected with older ActiGraph models. However, studies in older adults comparing the different generations of ActiGraph accelerometers have yet to be undertaken. As MVPA is positively associated with many health outcomes in older adults and recommended by the WHO [3], this study focussed on derivation of an MVPA cut point for older adults. While vigorous PA is not typically assessed in studies on older adults [24], future studies may wish to explore sedentary behaviour and vigorous PA cut points based on individual RMR.

## Conclusions

The MVPA cut points determined in this study suggest older adults are likely to have higher activity levels than reported by observational studies using younger adult MVPA cut points. This has important implications for accelerometer-based studies evaluating the relationships between chronic diseases and activity levels in older adults. It may also partly explain self-reported PA in older adult studies typically reporting higher levels of walking than that measured objectively via accelerometry using adult cut points [48, 49]. The high inter-individual variability in older adults’ MVPA cut points, partly explained by BMI, indicates that individual specific cut points, rather than mean values, should be applied when feasible, especially when a more accurate assessment of PA intensity and energy expenditure is important. Alternatively, future studies need to identify personal characteristics responsible for the high level of inter-individual variability in MVPA cut points for mobile older adults. As for walking at a self-selected moderately hard intensity in older adults with mobility limitations [23], these characteristics could be included in calibration equations to assist the determination of individual-specific cut points without performing calibration trials.

## Abbreviations

AIC: Akaike information criterion
BMI: Body mass index
LM models: Linear mixed models
MET: Metabolic equivalent
MVPA: Moderate to vigorous physical activity
PA: Physical activity
RMR: Resting metabolic rate
ROC: Receiver operating characteristics
VA: Vertical axis
VM: Vector magnitude
